# Enhancing Antitumor Efficacy of MUC1 mRNA Nano-Vaccine by CTLA-4 siRNA-Mediated Immune Checkpoint Modulation in Triple Negative Breast Cancer Mice Model

**DOI:** 10.3390/ijms26178448

**Published:** 2025-08-30

**Authors:** Amir Monfaredan, Sena Şen, Nahideh Karimian Fathi, Didem Taştekin, Alaviyehsadat Hosseininasab, Hamza Uğur Bozbey, Oral Öncül

**Affiliations:** 1 Department of Molecular Medicine, School of Advanced Technologies in Medicine, Tehran University of Medical Sciences, Tehran 1416634793, Iran; monfaredanamir@gmail.com; 2 Department of Basic Oncology, Oncology Institute, Istanbul University, Istanbul 34093, Türkiye; sena.sen@istanbul.edu.tr; 3 Genetics Research Center, University of Social Welfare and Rehabilitation Sciences, Tehran 1416634793, Iran; nahidkarimian@gmail.com; 4 Department of Clinic Oncology, Oncology Institute, Istanbul University, Istanbul 34093, Türkiye; didem.tastekin@istanbul.edu.tr (D.T.); ugurbozbey@yahoo.com (H.U.B.); 5 GeneDia Life Science Company, Tehran 1416634793, Iran; shrzd.hsni@gmail.com; 6 Department of Infectious Diseases and Clinical Microbiology, Internal Medicine, Istanbul Faculty of Medicine, Istanbul University, Istanbul 34093, Türkiye

**Keywords:** cancer immunotherapy, CTLA-4 siRNA, exosome-lipid nanoparticles, mRNA vaccine, triple-negative breast cancer (TNBC)

## Abstract

Immunotherapy, particularly approaches that combine tumor-specific vaccines with immune checkpoint modulation, represents a promising strategy for overcoming tumor immune evasion. While most mRNA-based cancer vaccines focus solely on antigen delivery, there is a need for platforms that simultaneously enhance antigen presentation and modulate the tumor microenvironment to increase therapeutic efficacy. This study presents a novel dual-nanolipid exosome (NLE) platform that simultaneously delivers MUC1 mRNA and CTLA-4-targeted siRNA in a single system. These endogenous lipid-based nanoparticles are structurally designed to mimic exosomes and are modified with mannose to enable selective targeting to dendritic cells (DCs) via mannose receptors. The platform was evaluated both in vitro and in vivo in terms of mRNA encapsulation efficiency, nanoparticle stability, and uptake by DCs. The co-delivery platform significantly enhanced antitumor immune responses compared to monotherapies. Flow cytometry revealed a notable increase in tumor-infiltrating CD8^+^ T cells (*p* < 0.01), and ELISPOT assays showed elevated IFN-γ production upon MUC1-specific stimulation. In vivo CTL assays demonstrated enhanced MUC1-specific cytotoxicity. Combined therapy resulted in immune response enhancement compared to vaccine or CTLA-4 siRNA alone. The NLE platform exhibited favorable biodistribution and low systemic toxicity. By combining targeted delivery of dendritic cells, immune checkpoint gene silencing, and efficient antigen expression in a biomimetic nanoparticle system, this study represents a significant advance over current immunotherapy strategies. The NLE platform shows strong potential as a modular and safe approach for RNA-based cancer immunotherapy.

## 1. Introduction

Breast cancer is the most common malignancy in women worldwide, and metastases develop in around 30% of cases [1]. Among its subtypes, triple-negative breast cancer (TNBC) remains a major clinical challenge as it lacks estrogen receptor (ER), progesterone receptor (PR), and human epidermal growth factor receptor 2 (HER2) [2,3]. In contrast to hormone receptor-positive or HER2-amplified tumors, there are no effective targeted therapies for TNBC, which are usually treated with cytotoxic chemotherapy [3]. PD-L1 expression is observed in approximately 20–30% of TNBC cases and is correlated with histological grade and lymphocyte infiltration [4]. Several clinical trials have evaluated immune checkpoint inhibitors in TNBC, including pembrolizumab (KEYNOTE-012, KEY-55 NOTE-355) [5,6], JS001 (NCT02838823) [7], atezolizumab (NCT01375842, IMpassion130) [8,9], and avelumab (JAVELIN) [10]. While favorable responses have been reported, particularly in PD-L1-positive patients, subsequent studies such as IMpassion131 have resulted in the withdrawal of certain regulatory approvals [9]. CTLA-4 has also been linked to TNBC progression, with higher expression levels reported in metastatic lymph node tissues [11]; however, no CTLA-4 inhibitors have yet been specifically approved for TNBC treatment [12]. The inherent heterogeneity, early metastatic potential, and mechanisms of therapy resistance emphasize the urgent need for biomarker-driven and innovative therapeutic strategies [13]. Cancer vaccines targeting tumor-associated antigens (TAAs) have proven to be a promising approach [14].

Cytotoxic T lymphocytes (CTLs) play a key role in tumor surveillance, but their activity can be suppressed by immune checkpoints such as CTLA-4, which inhibits early T cell activation by binding to B7 molecules on antigen-presenting cells [15,16,17,18,19,20]. CTLA-4 blockade has shown promise in cancer immunotherapy, yet systemic toxicity remains a concern. Mucin-1 (MUC1) is a tumor-associated antigen that is overexpressed and abnormally glycosylated in many cancers, contributing to immune evasion and tumor progression [2,21,22,23,24,25]. mRNA-based vaccines targeting MUC1 aim to induce tumor-specific immune responses, while siRNA-mediated silencing of CTLA-4 offers a more targeted approach to immune modulation. Despite the individual potential of MUC1 mRNA vaccines and CTLA-4 siRNA therapies, their combined application has not been extensively studied. This study hypothesizes that co-delivery of MUC1 mRNA and CTLA-4 siRNA via a nanolipid exosome platform could synergistically enhance antitumor immunity by promoting T cell activation and overcoming immune suppression in TBNC.

RNA-based vaccines represent a novel immunization strategy that enables the endogenous synthesis of antigenic proteins [26]. In contrast to conventional vaccines, they are based on mRNA or self-amplifying RNA (saRNA) encoding the target antigen and offer a rapid, scalable, and customizable approach [27]. Compared to peptide, carbohydrate, and DNA vaccines, mRNA vaccines offer several advantages. They are translated directly in the cytoplasm, bypassing entry into the nucleus, and achieve higher protein expression in less time [28]. In addition, the use of RNA avoids the risk of genomic integration and ensures transient expression, which increases safety [29]. Unlike peptide vaccines that often encode a single epitope, mRNA can encode multiple epitopes from the same sequence [30].

Despite these advantages, mRNA is unstable under physiological conditions, and its transfer into the cytosol of dendritic cells remains a challenge [29]. To overcome these limitations, nanoparticle-based delivery systems have been developed to improve stability, shelf life, and expression rates [31]. Among these, nanolipid–exosome hybrids have emerged as a new class of intracellular delivery systems [32]. Studies have shown that Nano-Lipid/exosome (NLE) can efficiently transport cargo into the cytoplasm and support mRNA/miRNA expression [33]. Surface modification of these particles with mannose ligands allows targeting to mannose receptors expressed on dendritic cells, facilitating cytosolic transfer and promoting CTL responses through MHC-I presentation [34]. A previous study demonstrated the efficacy of an mRNA vaccine targeting MUC1 delivered via a lipid-based carrier in a TNBC model, reporting enhanced tumor-specific immune responses and inhibition of tumor growth [35]. While this pioneering work validated the therapeutic potential of MUC1 mRNA vaccination, it did not combine immune checkpoint modulation on the same platform. Building upon this, our study introduces a dual-delivery system incorporating both MUC1 mRNA and CTLA-4 siRNA, enabling concurrent antigen presentation and immune checkpoint silencing in a dendritic cell-targeted NLE platform. To our knowledge, such a combinatorial and DC-targeted delivery approach has not been previously reported in the context of TNBC immunotherapy.

In this study, a multifunctional mRNA/siRNA nano-vaccine platform for the treatment of TNBC supported by a novel hybrid carrier system was developed and evaluated. The platform is based on the co-administration of mRNA encoding the tumor-associated antigen MUC1 and small interfering RNA (siRNA) targeting the immune checkpoint molecule CTLA-4 using synthetically engineered NLEs. The main objective is to elicit a robust, antigen-specific CTL response by in situ translation of MUC1 while suppressing CTLA-4 expression to attenuate T cell inhibition and promote durable antitumor immunity. The uniqueness of this study compared to existing approaches in the literature lies in the unique design of the delivery system. While previous studies have predominantly used either lipid nanoparticles (LNPs) or native exosomes, the present approach combines the biocompatibility and intracellular delivery capacity of exosomes with the structural stability and targeting accuracy of synthetic nanolipids, resulting in an advanced hybrid nanoparticle formulation. To improve cellular targeting, the surface of the NLEs was modified with mannose ligands recognized by mannose receptors, which are abundant on dendritic cells. This modification facilitated the efficient cytosolic transport of mRNA and siRNA payloads into antigen-presenting cells, thereby enhancing antigen presentation and subsequent T cell activation. The simultaneous administration of MUC1 mRNA and CTLA-4-targeting siRNA represents a dual immunotherapeutic strategy aimed at overcoming the immunologically “cold” tumor microenvironment characteristic of TNBC, a context in which such combined approaches have been explored only to a limited extent. The platform was evaluated through extensive in vitro and in vivo analyses in a murine TNBC model, focusing on physicochemical properties, transfusion efficiency, immunomodulatory capacity, and therapeutic efficacy. Importantly, the formulation is suitable for systemic administration, which enhances its translational potential for future preclinical and clinical applications. Overall, this work presents a synergistic and innovative immunotherapeutic strategy against aggressive and treatment-resistant TNBC.

## 2. Results

### 2.1. Characterization of mRNA-Containing NLE, Coding MUC1 and HA-Taq

**Figure 1:**
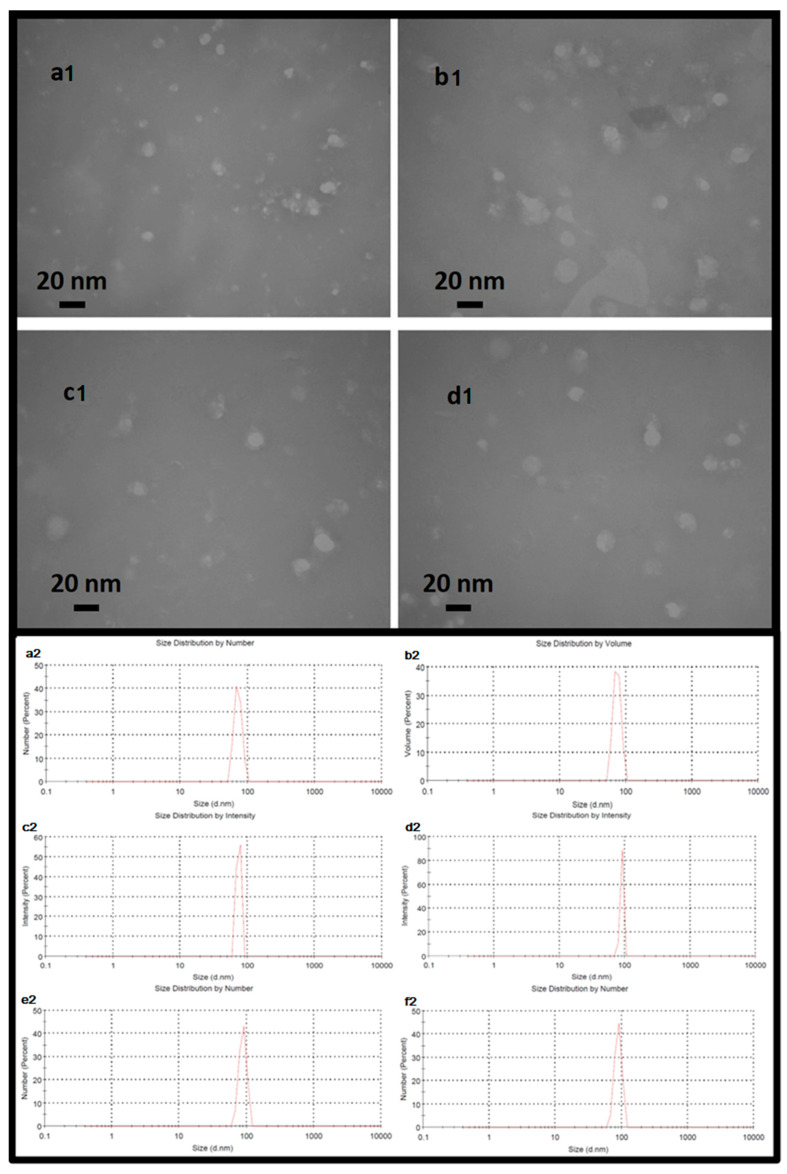
**Upper**: Nanoparticles imaged using the transmission electron microscopy method. The dimensions of the nanoparticles shown in the image indicate a range of approximately 16 to 20 nanometers for the central core of the nanoparticle. Exosomal particles (**a1**) coated with nanolipid (**b1**), exosomal particles containing mRNA (**c1**) and exosomal particles containing mRNA and coated with nanolipid (**d1**). **Lower**: The hydrodynamic size of exosomal particles (**a2**) coated with nanolipid (**b2**), exosomal particles containing mRNA (**c2**) and exosomal particles containing mRNA coated with nanolipid (**d2**), exosomal particles containing mRNA plus siRNA (**e2**), and exosomal particles containing mRNA plus siRNA coated with nanolipid (**f2**). The uniform dimensions and remarkable homology of each nanoparticle are clearly evident.

MUC1 is a transmembrane glycoprotein that is normally expressed on the apical surface of the ductal epithelium. To distinguish between exogenously and endogenously expressed MUC1 in tumor cells and normal tissue, a hemagglutinin (HA) tag coding sequence has been developed in various studies. At the 30th terminal of the MUC1 mRNA. The Kozak consensus sequence plays an important role in the initiation of the translation process. The untranslated region (UTR), including the Kozak sequence (GCCACC), was designed at the 50-terminus of MUC1 mRNA. RNA encoding MUC1 and the HA tag was synthesized using the in vitro transcription system. To confer desirable properties to the mRNA, such as increased stability against nucleases, increased translation, or reduced stimulation of the innate immune system, modified ribonucleotides were used to synthesize the RNA. Various studies have shown that NLE can efficiently encapsulate nucleic acids and peptides that could be used for the mRNA vaccine. The mRNA-loaded NLE was prepared in a water-in-oil microemulsion. The high PEG density on the NLE surface significantly increased the in vivo colloidal stability of the NP and thus improved the pharmacokinetic and pharmacodynamic profiles of the therapeutics. The surface of the NLE was modified with mannose to target the mannose receptor, which is highly expressed on DCs. The encapsulation efficiency of mRNA in NLE was about 89.5%. The CaP cores and the final NLEs were about 14 nm and 23 nm in diameter, respectively, as determined by TEM (Figure 1(a1,b1,c1,d1)).

The physical characterization of the exosomal formulations was evaluated by dynamic light scattering (DLS) to determine their hydrodynamic diameter and distribution profiles (Figure 1(a2,b2,c2,d2,e2,f2)). Exosomes loaded with siRNA (ExoSi) and complexed with ionizable low molecular weight cationic lipids (~5–10 kDa) formed nanoscale vesicles with uniform sizes between 70 and 98 nm, as previously described. Each 10^8^ exosomes was treated, on average, with 1 µg of siRNA. The exact size of these complexes was influenced by the preparation procedure. Complementary INSIGHT measurements confirmed these results and showed that the ExoSi ICL complexes had an average diameter of ~71 nm and a zeta potential of approximately +25 mV, reflecting the contribution of cationic lipid surface charge.

Further formulation of these complexes with ICLs of similar size resulted in slightly larger NLE particles, as expected ((Figure 1(a2,b2,c2,d2,e2,f2)). The NLEs exhibited characteristics of a hybrid vesicle system, composed of approximately 90% endogenous exosomal membrane and 10% synthetic lipids (a 9:1 ratio), consistent with the intended. Exosomes containing only mRNA (Figure 1(b2)) or mRNA in complex with siRNA (Figure 1(e2)) exhibited narrow, monodisperse size distributions. Addition of a nanolipid coating to mRNA- or mRNA/siRNA-loaded exosomes (Figure 1(c2,f2)) resulted in a modest increase in hydrodynamic size, suggesting successful surface modification. Remarkably, the DLS data also indicate a tendency of the ExoSi complexes to form transient aggregates, possibly explaining the occasional shifts to slightly smaller size values (Figure 1(a2,e2)), consistent with the particle size ordering observed in the INSIGHT analyses. Under all conditions, the nanoparticles exhibited favorable biophysical properties—uniform size distribution, low polydispersity, and hydrodynamic size in the range of 70–150 nm—highlighting their stability and suitability for systemic delivery and cellular uptake in therapeutic applications.

**Figure 2:**
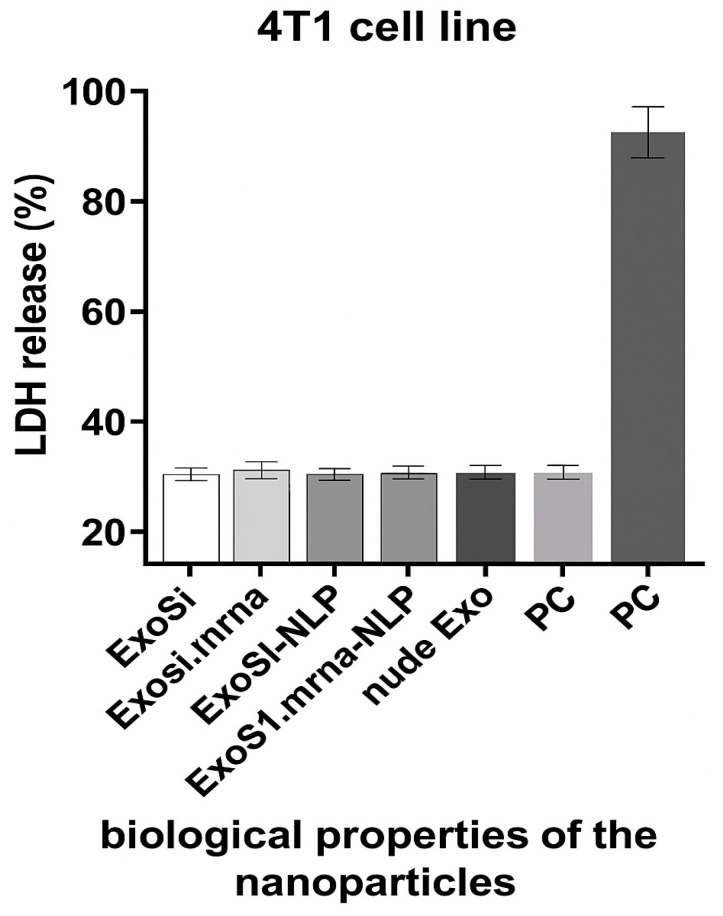
LDH release assay showing in vitro cytotoxicity of different nanoparticle formulations after 24 h incubation with 4T1 cells. All groups (ExoSi, ExoSi.mRNA, ExoSi.mRNA-NLP, and naked exosomes) exhibited LDH levels comparable to the negative control (NC), whereas the positive control (PC; 1% Triton X-100) showed significantly higher release, indicating minimal membrane disruption by the tested formulations.

The cytocompatibility of the nanoparticle formulations was evaluated using a lactate dehydrogenase (LDH) cytotoxicity assay (LDH Cytotoxicity Detection Kit, Roche Diagnostics) according to the manufacturer’s instructions. Mouse 4T1 breast cancer cells were seeded in 96-well plates at a density of 1 × 10^4^ cells/well and incubated overnight at 37 °C in a humidified atmosphere containing 5% CO_2_. The cells were then treated with the nanoparticle formulations or the controls for 24 h. The negative control (NC) consisted of untreated cells incubated in standard culture medium, while the positive control (PC) group was treated with 1% Triton X-100 to induce maximum LDH release. After the incubation period, the culture supernatant was collected and transferred to a new 96-well plate. LDH activity was quantified by measuring absorbance at 490 nm using a microplate reader. All treatments were performed in triplicate, and results were expressed as a percentage of total LDH release (Figure 2).

### 2.2. Exosomes Flowcytometry

Exosomes were incubated with 5000 antibody-coated beads in 250 μL PBS containing 2% casein for 10 h in a 10 mL tube without agitation at room temperature. After the binding step, the beads were stained with PE-conjugated anti-CD63 (clone TEA3/18 Im-munostep, S.L., Salamanca, Spain). After antibody binding, the beads were washed with filtered PBS containing 2% BSA and were stained with a Magnetic Rack (Magne Sphere(R) Mag. Sep. Stand 12-hole, 12 × 75 mm (Promega, Fitchburg, WI, USA, Ref Z5343). Streptavidin-PE (Immunostep, S.L., Salamanca, Spain) was then incubated, and the beads were subsequently washed with PBS-2% BSA. Samples were analyzed using FACSCalibur flow cytometers (Becton Dickin-son, Franklin Lakes, NJ, USA), and data were analyzed using Kaluza (Beckman Coulter, Singapore).

**Figure 3:**
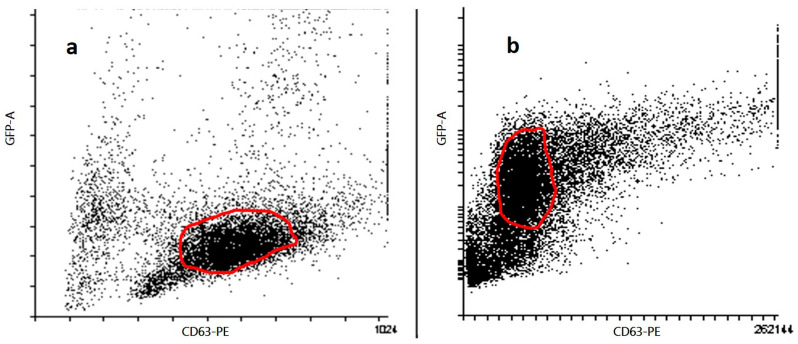
Characterization of exosomes by flow cytometry. Flow cytometric analysis of exosomes isolated from 4T1 cell culture supernatants (n = 3 biological replicates). (**a**) Exosomes stained with anti-CD63-PE antibody were gated based on forward and side scatter and CD63-PE positivity. (**b**) Exosomes from cells transfected with CD63-GFP construct were gated for GFP and CD63 expression. Data represent one representative dot plot from three independent experiments. Quantitative analysis and statistical comparisons were not included in this figure.

Flow cytometry identified a distinct CD63^+^GFP^+^ population (Figure 3), indicating that the exosomal marker CD63 and the GFP-labeled cargo were co-localized, consistent with successful cargo incorporation while maintaining vesicle integrity. In panel (a), exosomes stained with PE-conjugated anti-CD63 antibody show a distinct population with high CD63-PE signal, indicating successful labeling and confirming the presence of exosome-enriched vesicles. In panel (b), GFP-labeled CD63-positive exosomes show a comparable distribution in GFP and CD63-PE axes, confirming the expression of the CD63-GFP fusion protein and further supporting the identity of the vesicles as exosomes. The gated populations in both panels show consistent expression of CD63, a canonical tetraspanin marker for exosomes, confirming the successful isolation and characterization of the vesicular structures. These results confirm the integrity and exosomal nature of the vesicles used in the subsequent experiments.

### 2.3. Expression of MUC1 Fusion Protein with HA-Tag in Tumor-Derived Cell Line Stage IV Human Breast Cancer (4T1) and Lymph Node in Mice

**Figure 4:**
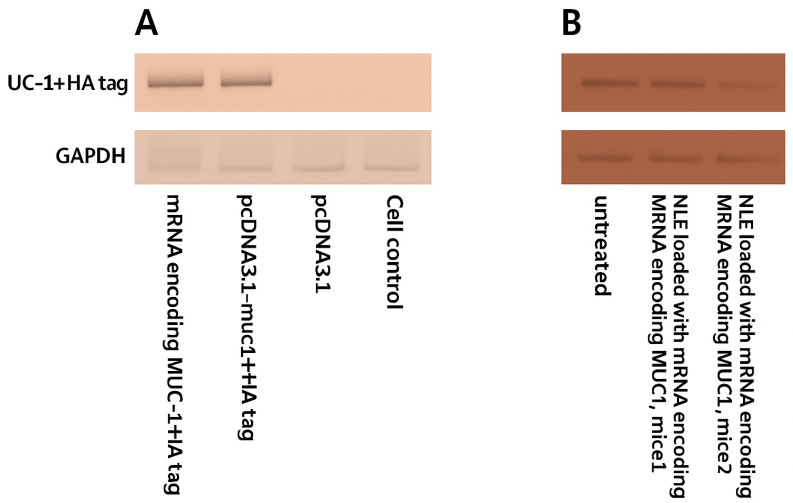
Expression of HA-tagged MUC1 fusion protein in 4T1 cells and lymph nodes of immunized mice. (**A**) Western blot analysis of MUC1-HA protein expression in 4T1 murine breast cancer cells 48 h after transfection with either mRNA encoding MUC1 + HA tag or plasmid pcDNA3.1-muc1 + HA. GAPDH was used as a loading control. (**B**) Western blot analysis of lymph node samples collected from immunized BALB/c mice (n = 3 per group) 48 h after subcutaneous injection of neutral lipid emulsions (NLE) loaded with mRNA encoding MUC1 + HA tag. Untreated lymph node samples served as negative controls. Representative bands from each animal are shown. Densitometric quantification and statistical comparison were not performed for these samples.

To distinguish exogenous from endogenous expression of MUC1, we synthesized an HA-tagged MUC1 gene. The recombinant plasmid and the transcribed mRNA encoding MUC1 and the HA tag were transfected into the 4T1 cell line, respectively. The expression of the MUC1 fusion protein was detected by a Western blot test with a peroxidase-labeled anti-HA antibody. The result showed that the HA tag was expressed in 4T1 cells after transfection. Since the HA tag was co-expressed with MUC1 and the HA tag was localized downstream of MUC1, Western blot analysis showed that exogenous MUC1 was successfully expressed in 4T1 cells (Figure 4A). Draining lymph nodes were harvested from mice immunized on day 10 after vaccination with NLE loaded with mRNA encoding the MUC1 fusion protein. Western blot analysis detected HA-tagged MUC1, indicating that NLE was able to release the mRNA into the lymph nodes and the mRNA was correctly translated into the target protein (Figure 4B).

### 2.4. In Vivo CTL Assay

**Figure 5:**
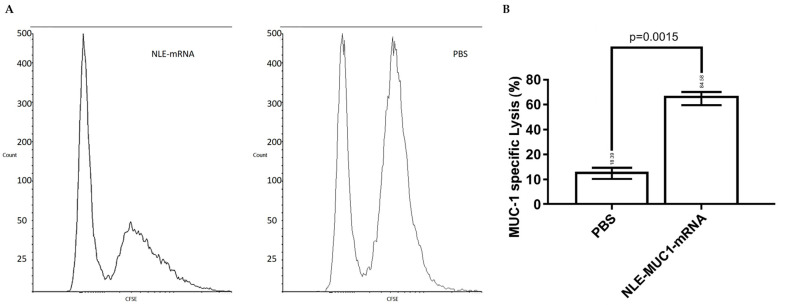
In vivo cytotoxic T lymphocyte response after vaccination. (**A**) In vivo CTL assay was performed to evaluate the cytotoxic activity induced by different treatments. Splenocytes from naïve mice were pulsed with 4T1 cell lysates transfected with NLE-MUC1 mRNA and labeled with a high concentration of carboxyfluorescein succinimidyl ester (CFSE^high^). Control cells pulsed with non-transfected lysate were labeled with a low concentration of CFSE (CFSE^low^). An equal number of CFSE^high^ and CFSE^low^ cells were mixed and intravenously injected into vaccinated or control mice. After 24 h, splenocytes were isolated from the spleens of recipient mice and analyzed by flow cytometry. (**B**) The percentage of specific lysis was calculated based on the relative reduction in CFSE^high^ cells, as described in the Materials and Methods section. Data are presented as mean ± SD (n = 3). *p* = 0.0015, unpaired *t*-test.

**Figure 6:**
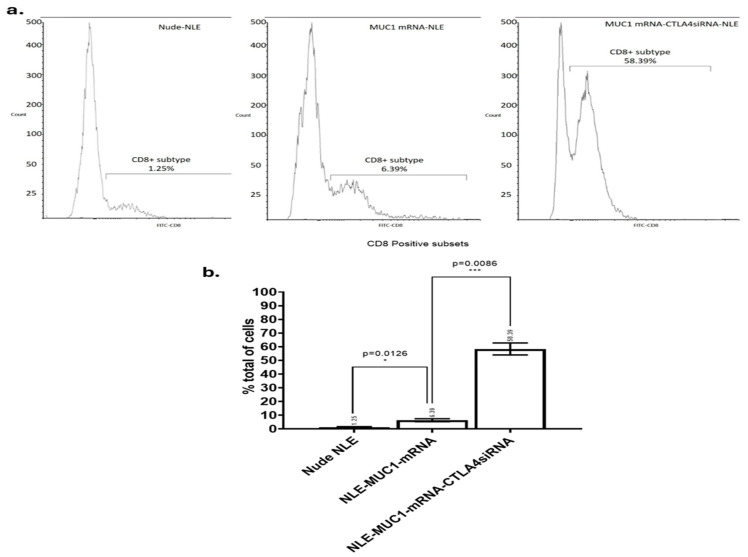
Flow cytometric analysis of tumor-infiltrating CD8^+^ T cells. (**a**) Tumor tissues were harvested on day 30 post-treatment and enzymatically dissociated into single-cell suspensions for flow cytometry. CD8^+^ T cell populations were identified using FITC-conjugated anti-CD8 antibodies. The percentage of CD8^+^ T cells among total tumor-infiltrating cells is shown for each treatment group: (**b**) Nude-NLE, NLE-MUC1 mRNA, and NLE-MUC1 mRNA + CTLA-4 siRNA (n = 3 mice per group). Data are presented as mean ± standard deviation (SD). Statistical comparisons were performed using one-way ANOVA followed by Tukey’s post hoc test. * *p* < 0.05, *** *p* < 0.01.

To assess the antigen-specific cytotoxic activity induced by the NLE-based mRNA vaccine, an in vivo CTL assay was conducted (Figure 5). Splenocytes obtained from naïve mice were pulsed with 4T1 cell lysates transfected with MUC1 mRNA and labeled with a high concentration (5 µM) of CFSE (CFSE^high^ cells). As a control population, splenocytes pulsed with non-transfected lysate were labeled with a lower concentration of CFSE (0.5 µM) (CFSE^low^ cells). These concentrations were selected based on the commonly used ranges that have been shown to preserve cell viability and function, as confirmed by preliminary titration assays [36]. An equal number of CFSE^high^ and CFSE^low^ cells were mixed and intravenously injected into immunized recipient mice on day 10 post-vaccination. As shown in Figure 6, a significant reduction in CFSE^high^ cells was observed in mice immunized with the NLE-based mRNA vaccine compared to PBS-treated controls, confirming the induction of robust antigen-specific CTL responses.

### 2.5. CD8^+^ Antitumor T Cells Infiltration Analyzed by FlowCytometry Assay

As shown in Figure 6, the vaccine group, the CTLA-4 siRNA-containing NLE group and the combination group all induced a significant increase in tumor-infiltrating CD8^+^ T cells compared to the PBS, naked mRNA, empty NLE, and isotype control groups. The combination group induced significantly more tumor-infiltrating CD8^+^ T cells than the vaccine and CTLA-4 siRNA-containing NLE groups. The result showed that although treatment with the MUC1 vaccine or CTLA-4 siRNA-containing NLE could increase the number of CD8^+^ T cells infiltrating the tumor, the combination therapy was most effective in promoting the infiltration of CD8^+^ T cells compared to the individual treatments.

### 2.6. Production of IFN-g by Lymphocytes from Vaccinated Mice

**Figure 7:**
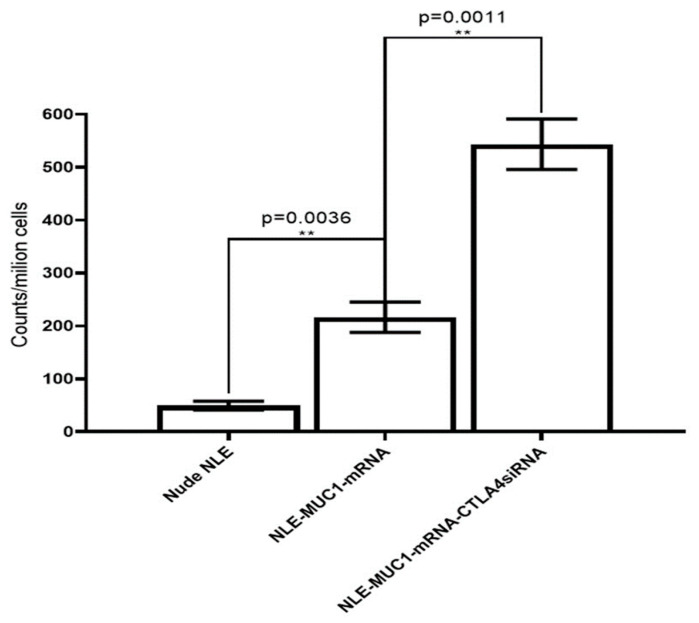
Production of mouse IFN-g was detected by ELISPOT assay. Representative graph of the IFN-g ELISPOT assay. Spleens were harvested from each treated mouse for 10 days, and single cell suspensions were stimulated for 24 h with either cell lysates transfected with MUC1 mRNA. Production of IFN-g was detected using the BD ELISPOT substrate set. Quantitation of the ELISPOT assay. Data are shown as mean ± SD. ** *p* < 0.01.

Tumor antigen-induced IFN-g production was measured with a BD ELISPOT assay system (Figure 7). Spleens were harvested sterile from each treated mouse 10 days after immunization and separated into single cell suspensions. Cells were stimulated with either 2 mg/mL of cell lysates transfected with MUC1 mRNA or with MCF7 cell lysates as a control. The production of IFN-g was detected using a BD ELISPOT substrate set. Positive reactions were counted manually. The data showed that only MUC1-specific induction produced IFN-g. Combined therapy with CTLA-4 siRNA containing NLE and vaccine can improve immune response enhancement MUC1 is a widely used target for antitumor immunotherapy. However, an NLE-based RNA vaccine with MUC1-encoding mRNA in the central core and an asymmetric endogenous lipid outer membrane for TNBC therapy is a novel approach. An in vivo CTL assay and an IFN-g production assay showed that an MUC1 vaccine can induce antigen-specific antitumor immunity. However, activation of T cells by the T cell receptor and CD28 leads to increased expression of CTLA-4, an inhibitory receptor for B7, and eventually, the inhibitory pathway attenuates and terminates T cell responses. To enhance T cell function and improve vaccine efficacy, the NP-based mRNA vaccine was combined with a CTLA-4 siRNA-containing NLE for TNBC therapy. As shown in Figure 7, both the MUC1 vaccine group (*p* < 0.01) showed strong antitumor activity. The suppression of CTLA with siRNA potentiated the efficacy of the MUC1 vaccine, which showed superior inhibitory activity compared to the vaccine group (*p* < 0.01). The result suggests that the MUC1 vaccine can induce an antitumor immune response and that the therapeutic effect of the vaccine on TNBC is significantly enhanced by additional treatment with CTLA-4 siRNA containing NLE. It is noteworthy that the endogenous expression level of MUC1 on 4T1 cells successfully induced an anti-gene-specific T cell response, leading to immune response enhancement, although the lysate of naïve 4T1 cells could not stimulate splenocytes for antigen presentation in the in vivo CTL assay or induce the production of IFN-g in vitro.

## 3. Discussion

The lack of targeted therapies and the poor prognosis of patients with TNBC have strongly encouraged the need to discover molecular targets and develop new therapeutic approaches. Immunotherapy slows the progression of TNBC and represents an attractive approach for the treatment of TNBC. In recent years, mRNA-based vaccine platforms have attracted considerable attention, especially after the SARS-CoV-2 pandemic, due to their advantages such as strong immunogenicity, ease of production, and rapid development capability [37,38]. The success of mRNA vaccines largely depends on the carrier systems used. In the current literature, LNPs, polymeric nanoparticles, and lipoplex systems are frequently used [39,40]. However, most of these systems have certain limitations in terms of target specificity, recognition by immune cells, and toxicity. In this study, a nanolipid exosome-like system was developed, which differs from similar strategies described in the literature. Unlike traditional liposomal or exosomal systems, our NLE vesicles combine endogenous exosomal membranes (~90%) with synthetic lipid components (~10%), offering improved targeting capacity and membrane stability.

In this study, NLE was used as a carrier and adjuvant system for the development of an mRNA vaccine encoding the tumor-associated antigen MUC1. In addition, the vaccine was combined with CTLA-4 siRNA containing NLE to enhance the vaccine’s immune response against TNBC by improving T cell function. In vivo studies showed that the NP-based mRNA vaccine modified with a mannose targeting the DC surface receptor could efflux into the lymph node and that the encapsulated mRNA could be successfully expressed in the DCs of the lymph node. Furthermore, in vivo studies showed that the NP vaccine could elicit a strong, antigen-specific CTL response against 4T1 TNBC cells and that combined treatment with a CTLA-4-siRNA-containing NLE could significantly enhance the anti-tumor immune response compared to the vaccine or CTLA-4-siRNA alone. This synergistic effect supports the growing evidence that co-administration of tumor antigens and immune checkpoint inhibitors can turn ‘cold’ tumors into ‘hot’ tumors by enabling robust T cell infiltration and activation [41,42]. Our siRNA-based checkpoint inhibition strategy may offer advantages over monoclonal antibodies in terms of reduced systemic toxicity and improved intracellular silencing efficiency. Our siRNA-based checkpoint inhibition strategy may offer advantages over monoclonal antibodies in terms of reduced systemic toxicity and improved intracellular silencing efficiency. Our results are consistent with those of Lin et al. [35], who demonstrated that co-delivery of MUC1 mRNA and CTLA-4 blockade can activate antigen-presenting cells and suppress tumor progression in a triple-negative breast cancer (TNBC) model. However, our study advances this strategy by utilizing a novel nanolipid–exosome hybrid (NLE) platform for the co-delivery of MUC1 mRNA and CTLA-4 siRNA. This bioinspired system offers improved biocompatibility, efficient DC-targeting, and enhanced endosomal escape. As a result, we observed stronger activation of CD8^+^ T cells and a more immunostimulatory tumor microenvironment, including elevated IFN-γ secretion and increased infiltration of tumor-infiltrating lymphocytes. These findings suggest that the use of exosome-based nanocarriers may provide superior therapeutic efficacy compared to conventional liposomal systems. Although our study did not include an irrelevant antigen or scrambled siRNA control group, several experimental results argue against nonspecific immune activation. In particular, the immune response (CD8^+^ T cell infiltration, CTL activity, IFN-γ production) was significantly increased only in the groups receiving both MUC1 mRNA and CTLA-4 siRNA and not in the groups treated with PBS, empty NLE, or mRNA alone. In addition, ELISPOT analysis showed that IFN-γ secretion was induced exclusively when stimulated with MUC1-transfected lysates and not with lysates from irrelevant cell lines, emphasizing antigen specificity. Overall, these results indicate that the observed immune activation is primarily due to the specific immunogenic content of the vaccine and not to non-specific effects of the carrier.

TEM analysis confirmed the successful formation and structural integrity of the synthesized nanoparticles. As shown in Figure 1, the calcium phosphate cores had an average diameter of about 14 nm (Figure 1(a1)), while the fully assembled nanolipid-based exosome-like particles reached a diameter of about 23 nm after lipid coating and mRNA encapsulation (Figure 1(b1,c1,d1)). These observations are consistent with previous reports demonstrating the ability of NLEs to form stable nanoscale carriers for nucleic acid transport [43]. The use of modified ribonucleotides during in vitro transcription conferred greater stability to mRNA, protected it from enzymatic degradation, and improved translation efficiency, which is critical for mRNA therapeutics [37]. In addition, the high polyethylene glycol (PEG) density on the surface of the NLEs likely contributed to improved colloidal stability and prolonged systemic circulation, as shown by pharmacokinetic studies with PEGylated nanoparticles [44]. Decoration of the NLE surface with mannose enabled additional targeting by utilizing the high expression of mannose receptors on DCs, which facilitated efficient uptake and antigen presentation [34]. While mRNA encapsulation efficiency in the literature is generally between 60% and 80%, an encapsulation yield of approximately 89.5% was achieved in this study. This result indicates that the carrier provides strong protection for the mRNA and is an efficient system in terms of production [37,39]. In addition, the physical parameters obtained by DLS, TEM, and zeta potential analyses demonstrate the stability of the system with features such as low polydispersity and good colloidal stability. This type of characterization is either absent or limited in many previous studies. These results suggest that the formulated NLE system is a promising platform for mRNA-based vaccines or therapeutics, especially for applications requiring targeted delivery and enhanced intracellular expression. The NLE structure used in this study exhibits both the properties of lipid nanoparticles and the advantages of an exosome-like natural carrier architecture, which enables improved biocompatibility and intracellular targeting. In other systems, such as LNPs, PEGylation is usually performed; however, an exosome-like structure is generally not offered. Exosomal architectures allow more natural interactions in intracellular transport and antigen presentation processes [39,40]. In addition, mannose modification enabled targeting of dendritic cells, making the NLE system not only a means of transport but also an active component that can modulate the immune response. In previous studies, such targeted release was usually achieved with polyclonal binders or antibodies [45], whereas in this study, this function was achieved by using natural ligands such as mannose.

The use of tumor-specific mRNA as a vaccine is a focus of current research and has several advantages, including feasibility, applicability, safety, and efficacy in generating immune responses [46]. A variety of NPs have been investigated for effective gene delivery in vivo, including lipid- and polymer-based NPs in which the negatively charged nucleic acid molecules are encapsulated in the hydrophilic core or adsorbed on the cationic surface [47]. NLEs, which consist of a biological core and an asymmetric lipid bilayer, were first developed in our laboratory and used for the transport of siRNA and peptides. LDH release assay was performed to assess the cytocompatibility of NLE formulations. All nanoparticle groups, including siRNA- and mRNA-loaded exosomes with or without lipid coating, exhibited LDH levels below 20%, comparable to the negative control. These results indicate minimal membrane disruption and excellent in vitro biocompatibility. LDH release reflects acute membrane-associated toxicity, and therefore, additional studies will be required to evaluate long-term safety.

Although LDH results confirm negligible acute cytotoxicity, this assay does not capture potential long-term effects such as apoptosis, oxidative stress, or genotoxicity. Therefore, future studies with longer exposure duration and in vivo systems are needed to fully assess the biological safety profile of these nanoparticles [48,49]. Interestingly, although the nanoparticles did not cause measurable membrane damage, their biological activity and mode of action indicate that they were successfully internalized by the target cells. This is consistent with previous findings showing that exosomes and lipid-based nanocarriers can enter cells via non-disruptive, energy-dependent endocytic pathways, such as clathrin-mediated, caveolin-mediated, or macropinocytosis [50,51]. The ability to transport functional cargo without compromising membrane integrity supports the potential of the NLE system as a safe and effective transport platform for therapeutic applications. These results are consistent with previous studies showing that exosomes and PEGylated lipid nanoparticles exhibit low immunogenicity and do not affect cell viability during intracellular administration [52]. Furthermore, the similar response between the parental exosomes and the hybrid NLE formulations suggests that the processes of encapsulation and nanolipid modification do not alter the physicochemical stability or safety profile of the delivery vehicle. The absence of cytotoxicity, even during transfection of car-go-containing exosomes, emphasizes the potential of NLEs as a safe and effective system for the delivery of therapeutic nucleic acids. These results support the use of NLEs in in vivo applications where maintenance of host cell viability is critical for therapeutic efficacy. The DLS and IN-SIGHT analyzes confirmed the successful assembly and surface modification of the exosomal formulations, with a continuous increase in hydrodynamic diameter after nanolipid coating. The narrow size distributions and low polydispersity index values observed in all groups reflect good colloidal stability—an important prerequisite for systemic delivery. In particular, the shift in zeta potential after ICL complexation indicates efficient modulation of surface charge, which may improve cellular uptake and circulation time. These physical properties emphasize the structural integrity and delivery potential of the NLE system. Indeed, flow cytometric analysis confirmed the identity and purity of the isolated vesicles as exosomes by the consistent expression of CD63, a well-established exosomal surface marker. The high CD63-PE fluorescence intensity and the overlapping distribution of CD63-GFP confirmed both the antibody-based and genetic labeling strategies, indicating robust detection. These results confirm the reliability of the isolation protocol and ensure that the vesicles used in downstream applications are indeed of exosomal origin.

Surface modification with mannose targeting DC-specific receptors is consistent with findings in the literature reporting improved lymph node targeting and enhanced antigen presentation by mannose-conjugated carrier systems [53]. When the MUC1 mRNA was packaged into NLE, the RNA molecules were condensed and encapsulated into the exosomal core. An in vivo study showed that NLEs can successfully transport and release MUC1 mRNA into the cytoplasm of target cells in lymph nodes. In the present study, the NLE-based mRNA vaccine was developed for the treatment of TNBC. This means that NLE is also a powerful system for more tumor-specific RNA delivery. Many MUC1-based immunotherapies have been evaluated in clinical trials. Two MUC1-based therapeutic vaccines are in clinical development. These vaccines are long, non-glycosylated polypeptides derived from the VNTR sequence of MUC1. The successful expression of HA-tagged MUC1 in both transfected 4T1 cells and draining lymph nodes demonstrates that the mRNA vaccine construct was efficiently transferred and translated.

Importantly, the in vivo CTL assay revealed a significant antigen-specific cytotoxic T lymphocyte response in mice immunized with NLE-formulated MUC1 mRNA, confirming that the vaccine is capable of eliciting functional CD8^+^ T cell responses against tumor antigens [37]. Most of the potential CTL epitopes on the MUC1 molecule are outside the VNTR; therefore, it is important to incorporate the entire MUC1 molecule into therapeutic vaccines. Two such vaccines use poxviruses as vectors for the MUC1 gene sequence, but improved DC uptake and T-cell recruitment are considered necessary. MUC1 is abnormally overexpressed in a variety of epithelial tumors. It has also been shown to be expressed on the epithelial cells of various healthy tissues and to act as a protective lubricant during bacterial infections. Overexpressed MUC1 is hypo-glycosylated and not restricted to the cell surface. Tumor-infiltrating cytotoxic T cells can be inhibited by the co-inhibitory signal of CTLA-4. Blockade of CTLA-4 by antibodies has been shown to enhance the antitumor immune response in both preclinical models in mice and in clinical trials. Tumor inhibition was significantly more effective when vaccination with MUC1 mRNA-loaded NLE was combined with silencer blockade of CTLA-4. Increased T-cell infiltration of tumors was observed after simultaneous blockade of the CTLA-4 pathway. Our results are consistent with those observed in models using combined mRNA vaccines and immune checkpoint blockade, such as when using HA-labeled antigens and cargo-modifying transporters, which have shown promise for enhancing antigen-specific CD8^+^ responses. In addition, flow cytometric analysis showed increased infiltration of CD8^+^ T cells into the tumor microenvironment after treatment with either the MUC1 mRNA vaccine or CTLA-4 siRNA-loaded NLE, with the combination therapy showing the strongest effect. This is consistent with previous results suggesting that immune checkpoint blockade, together with an antigen-specific vaccination, may act synergistically to overcome T cell exhaustion and improve therapeutic efficacy [54]. While our study did not include direct phenotypic characterization of tumor-infiltrating CD8^+^ T cells for markers such as granzyme B, IFN-γ, PD-1, or exhaustion profiles, functional data from in vivo CTL assays and ELISPOT suggest that these T cells were actively involved in cytotoxic responses. The observed antigen-specific cytotoxicity and IFN-γ secretion suggest that the infiltrating CD8^+^ population was not only recruited but was functionally competent. Nevertheless, we acknowledge this limitation and consider detailed phenotypic profiling to be an important direction for future studies to fully define the activation and exhaustion status of tumor-infiltrating lymphocytes. The ELISPOT results also confirmed the immunogenicity of the vaccine, with increased IFN-γ production observed exclusively in response to MUC1-specific stimulation, emphasizing the specificity and efficacy of immune activation. This study not only demonstrated translation of the mRNA vaccine but also its multidimensional functional outcomes, including CD8^+^ T cell responses, IFN-γ production, tumor infiltration, and inhibition of tumor growth in the 4T1 tumor model. While most preclinical studies in the literature are limited to CD8^+^ activation or tumor growth monitoring, this study comprehensively analyzed the immune response at both cellular and functional levels using ELISPOT, flow cytometry, and in vivo tumor volume measurements [38,45]. Although no direct quantification of CTLA-4 mRNA or protein levels in immune cells was performed in this study, the observed immunological effects—such as increased tumor-infiltrating CD8^+^ T cells, enhanced IFN-γ secretion, and stronger CTL activity in the siRNA-treated groups—indirectly confirm the functional elimination of CTLA-4. These immune responses are consistent with effective checkpoint inhibition and reflect the downstream consequences of CTLA-4 suppression.

While most mRNA-based tumor vaccine studies in the literature focus exclusively on the administration of mRNA encoding tumor antigens [37,38], this study additionally used siRNA targeting CTLA-4, one of the major immunosuppressive mechanisms. Taken together, these data suggest that MUC1 mRNA-based vaccination, especially in combination with inhibition of the immune checkpoint CTLA-4, is a promising immunotherapeutic approach for the treatment of triple-negative breast cancer. Since this dual strategy targets both antigen presentation and immune regulation, it effectively boosts anti-tumor immunity and may provide a platform for future combinatorial mRNA-based cancer therapies. These results suggest that co-delivery platforms not only promote CTL-mediated cytotoxicity but also facilitate the generation of long-term memory T cells, a hypothesis that should be explored in future studies [55]. Various cancer vaccines in combination with blockade of inhibitory signaling pathways have been validated in preclinical models, and enhanced T cell infiltration of various tumors has been demonstrated following this combination therapy.

While the present study demonstrates the promising potential of an MUC1 mRNA-based vaccine in combination with CTLA-4 siRNA delivered via NLE platforms, several limitations should also be noted. First, although the in vivo mouse model provides valuable insights into immunogenicity and therapeutic response, it cannot fully reconstruct the complexity of the human tumor microenvironment, especially in TNBC. Second, long-term immunologic memory—including persistence and functionality of T cell responses—as well as potential off-target effects associated with repeated siRNA administration have not been investigated. Systemic toxicity following repeated administration also remains to be investigated. While mannose modification has been shown to improve targeting to dendritic cells, the broader specificity and biodistribution of NLEs in both immune and non-immune tissues remain to be systematically investigated. Although blockade of CTLA-4 with monoclonal antibodies has already been demonstrated [45], intracellular silencing using siRNA represents a significant innovation, offering the potential for more effective intracellular inhibition and reduced systemic toxicity compared to antibody-based approaches. Importantly, the effects of this co-delivery system on immunosuppressive cell populations such as myeloid-derived suppressor cells (MDSCs) and regulatory T cells (Tregs) remain unexplored. Beyond enhancing CD8^+^ T-cell infiltration, CTLA-4 silencing may influence additional immunoregulatory mechanisms within the tumor microenvironment (TME). CTLA-4 is a key mediator of regulatory T cell (Treg) suppressive activity via the CTLA-4/B7 axis, which maintains immune tolerance in the TME [12,16]. Previous studies have shown that CTLA-4 blockade reduces intratumoral FoxP3^+^ Tregs, thereby alleviating local immunosuppression [56]. Consequently, delivery of CTLA-4 siRNA through the NLE platform may not only activate effector T cells but also attenuate Treg-mediated suppression, resulting in a dual mechanism that promotes a more robust and durable antitumor response. These limitations emphasize the need for comprehensive mechanistic studies and advanced preclinical models to better evaluate the safety, specificity, and translational potential of this therapeutic approach. Future investigations should validate this hypothesis by quantifying intratumoral Treg frequency and phenotype after CTLA-4 silencing and correlating these findings with therapeutic outcomes. Comprehensive mechanistic studies and advanced preclinical models will be essential to confirm the safety, specificity, and translational potential of this strategy.

Beyond the immunological context, this work aligns with the emerging integration of nanotechnology and computational biology. While tools such as AlphaFold have transformed drug discovery through protein structure prediction and target identification, our study approaches the reverse challenge—function-driven optimization of nanocarrier–receptor interactions [57]. Mannose modification of NLEs exemplifies a rational design principle, leveraging receptor–ligand complementarity for targeted dendritic cell delivery. This strategy mirrors the “structure-to-function” paradigm in computational biology and suggests future opportunities for synergy between experimental design and AI-based modeling. For example, predicted receptor structures could guide ligand optimization, while molecular dynamics simulations and generative AI could facilitate the creation of modular nucleic acid carriers. Such convergence would enable iterative cycles of in silico design and experimental validation, accelerating the development of smart delivery systems for complex diseases.

In order for this strategy to be implemented in clinical practice, several crucial steps need to be taken in follow-up studies. A key focus will be to comprehensively investigate the safety profile and tolerability of the NLE platform, ideally in larger animal models than rodents. These assessments should include dose escalation protocols and repeated dosing to investigate biodistribution, systemic toxicity, and the risk of unintended immune responses. The clinical success of LNP-based mRNA vaccines, as used in the COVID-19 pandemic, is a valuable precedent for adapting similar delivery systems to cancer immunotherapy [37,52]. However, the combination of mRNA vaccines with siRNA to inhibit immune checkpoints brings additional challenges, particularly with regard to long-term immune regulation, the risk of T cell exhaustion, and the overall balance of the immune system.

Further data are needed to determine the optimal dosing strategy, the durability of immune responses, and the ability to generate memory T cells in early human studies. In particular, assessment of long-term immune memory and the phenotypic and functional properties of CD8^+^ and CD4^+^ T cells after treatment will be critical to understanding the potential for durable anti-tumor immunity [43]. Long-term monitoring of the immune system, including relapse responses and T-cell persistence, should be incorporated into future preclinical and clinical trial designs. It is important that the molecular heterogeneity of TNBC in humans, characterized by different intrinsic subtypes (e.g., basal-like, mesenchymal, immunomodulatory), variable immune infiltration patterns, and different mutational landscapes, is taken into account in clinical translation. Preclinical models may not fully reflect this heterogeneity, which could impact therapeutic response. Stratification of TNBC patients based on immunophenotypes or molecular biomarkers such as PD-L1 expression or tumor mutational burden could, therefore, improve the precision and efficacy of this therapeutic strategy. Studying the effects of this platform on the tumor microenvironment, especially using modern tools such as single-cell RNA sequencing or spatial transcriptomics, could provide important insights into the modulation of immune cell populations [28]. These technologies could shed light on how therapy reprograms cellular interactions within the tumor microenvironment and supports or inhibits anti-tumor immunity. Of particular interest is the role of immunosuppressive cells such as MDSCs and Tregs, which are known to suppress effective immune responses. The impact of this platform on the frequency, phenotype, and suppressive function of MDSCs and Tregs should be fully investigated to determine whether the therapeutic benefit results from enhanced effector responses, suppression of regulatory populations, or both.

Although local or tumor-targeted delivery of CTLA-4 siRNA via NLE-based nanoparticles may reduce systemic exposure, the systemic safety profile of CTLA-4 blockade by siRNA remains a concern. CTLA-4 plays a critical role in immune system homeostasis, particularly in regulating Treg function and preventing autoimmunity. Therefore, potential off-target effects, systemic immune activation, and the risk of autoimmune toxicities need to be carefully monitored in future studies. Preclinical safety assessments, including cytokine release profiles, autoimmune antibody screening, and histopathologic analysis of major organs, should be performed prior to clinical use. Manufacturing considerations, including GMP-compliant production and rigorous physicochemical characterization of NLE particles, such as mRNA encapsulation efficiency, particle size distribution, and batch reproducibility, are also essential prerequisites for clinical translation. In addition, stratification of patients based on immunological or molecular biomarkers could lead to personalized treatment strategies and increase the likelihood of clinical success. Given the encouraging preclinical results of this study, a phase I clinical trial with TNBC patients, ideally stratified according to immunological or molecular biomarkers, could be a sensible next step. Such a study would not only assess safety and tolerability but also provide insights into the immunological correlates of response and resistance, thus supporting the rational planning of subsequent clinical trials.

## 4. Materials and Methods

### 4.1. Cell Culture

The 4T1 TNBC cell line was acquired from the GeneDia Culture Collection. These cells were maintained in DMEM supplemented with 20% FBS and 1% penicillin/streptomycin at 37 °C in a humidified atmosphere with 5% CO_2_. Cultures were maintained in a humidified incubator at 37 °C and 5% CO_2_ and analyzed monthly for mycoplasma contamination using the PCR Mycoplasma Test Kit II (AppliChem, Darmstadt, Germany) according to the manufacturer’s instructions. For extraction of exosomes, 4T1 TNBC cells were first seeded in 15 cm dishes and treated with 0.1 mg/mL^−1^ PMA in medium for 24 h. The media were then replaced with RMPI medium 1640 containing 10 μg/mL^−1^ DSPE-PEG2000-Mal for 48 h. The remaining supernatant was collected after centrifugation at 2000 g for 15 min, followed by filtration through a 0.22 μm membrane. The filtrate was then concentrated using a 100 kDa ultrafiltration tube at 2200 g for 20 min to obtain the crude extract. Exosomes were then extracted from the crude extract using a Plasma/Serum Exosome Purification and RNA Isolation Mini Kit (Norgen Biotek Corp, Thorold, ON, Canada) according to the manufacturer’s protocols.

### 4.2. Intact Exosome Purification and Exosomal RNA Isolation Followed by Complementary DNA Synthesis

Exosomes were isolated and RNA was extracted using the Plasma/Serum Exosome Purification and RNA Isolation Mini Kit (Norgen Biotek Corp, ON, Canada) according to the manufacturer’s protocols. Non-coding total RNAs and small RNAs, such as miRNAs, were transferred to the cDNA extraction kit (ABM good Cat# G902). The miRNA sample was prepared by mixing 2 µL of 5× poly(A) polymerase reaction buffer, 1.5 µL ATP (10 mM), 1.5 µL MnCl_2_ (25 mM), 0.5 µL poly(A) polymerase, yeast (1 µg/µL), and 2.5 µL H_2_O. The mixture was then incubated at 37 °C for 30 min. Then, 2 µL of miRNA oligo (dT) adapter (10 mM) was added to the remaining material. The mixture was incubated at 65 °C for 5 min and then cooled on crushed ice. Finally, 1 µL of dNTPs (10 mM), 4 µL of 5× RT buffer, 1 µL of RTase (200 U/µL), and 2 µL of H_2_O were added to the above mixture. The cDNA synthesis was performed by incubating the samples for 15 min at 42 °C and 10 min at 70 °C.

### 4.3. Preparation of Exo-CTLA-4siRNA

Synthetic siRNAs were acquired from MWG (Metabion, Planegg, Germany) and had the following sequences: for the animal model, CTLA-4m-silencer: 5′ GCAGCAUAAGGAUAUAGCA 3′ (sense); for 4T1 TNBC line, CTLA-4h-silencer: 5′ GAGCUGAGGCAAUUCUAAC 3′ (sense). After 24 h of incubation, 0.1 nmol of unbound siRNA was exposed to 25 μL of human plasma. The resulting mixtures were subjected to electrophoresis on a 20% TBE PAGE gel at 90 V for 180 min in TBE running buffer. Following electrophoresis, the gel was treated with 2.5 μL of EVAgreen gold in 25 mL of TBE buffer for 30 min and then detected using Sage 6000.

For the preparation of Exo-CTLA-4siRNA, 0.5 nmol siRNA was added to 100 μL of exosomes (0.5 mg/mL^−1^), and then sonicated using an ultrasonic homogenizer (Sonica, Tehran, Iran) with the following settings: ultrasonic power 20 W, 10 cycles of 20 s and 100 s off. The mixture was then incubated for 30 min at 4 °C. After incubating the mixture for 24 h, 0.5 mg of siRNA was added, and the solution was then washed twice with 150 μL of PBS using a 100 kDa ultrafiltration tube to remove any unconjugated siRNA and unloaded siRNA.

### 4.4. Characterization of Exosomes

To observe the morphology of Exo-SiRNA and Exosome, transmission electron microscopy (TEM) was performed using a Hitachi microscope in Tehran, Iran. For this, 2 μL of engineered and nude exosomes were placed on a carbon-coated copper grid and allowed to dry for 10 min. Then, 20 μL of ammonium molybdate negative stain solution was added and left to dry for another 10 min. To ensure an appropriate concentration for measurement, the engineered exosomes were diluted with PBS. The primary antibody used was CD63, while the secondary antibody was anti-mouse IgG conjugated with horseradish peroxidase. The proteins were detected using Western blotting.

### 4.5. Lipid Nanoparticles (NLP) Containing Exo-siRNA Using Microfluidic Technology by INSIGHT Nano Synthesis

To achieve this, the total flow rate (TFR) control was manipulated between 2–20 mL/min, while the ILR (ionisable lipid reagent) concentrations (2–10 mg/mL) were varied. The aqueous solution to solvent flow rate ratio was then adjusted from 1:1 to 10:1, using 10 mg/mL ILR in 40% ethanol, while the TFR remained at 5 mL/min. The concentrations in our assay always refer to siRNA (1 μg/mL means 1 μg siRNA in 1 mL culture). The resulting NLP product was collected in a 15 mL falcon tube, and the initial volume of 0.5 mL and the final volume of 0.05 mL NP solution were discarded separately. After synthesizing the NANO-LIPID/EXOSOME (NLE), a solvent exchange procedure was used to remove the ethanol and replace it with water. The NANO-LIPID/EXOSOME (NLE) was then diluted and centrifuged three times at 1600× *g* for 30 min runs using ultra-centrifugal philtres with a nominal molecular weight limit of 10,000 NMWL. The size and distribution of NANO-LIPID/EXOSOME (NLE)s were measured in both distilled water and PBS using the Malvern Zetasiser. The final hybrid vesicles consisted of predominantly exosomal membrane components (~90%) combined with synthetic cationic lipids (~10%), forming a biomimetic bilayer with enhanced colloidal stability and targeting potential.

### 4.6. Nanoparticle Characterization

The Brookhaven ZetaPALS system (Brookhaven Instruments, Holtsville, NY, USA) was utilized to determine the particle sizes and zeta potentials through photon correlation spectroscopy (PCS) and phase analysis light scattering (PALS). The manufacturer’s software was used for data analysis, with a viscosity and refractive index of pure water at 25 °C applied. The complexes were analyzed for size in five separate runs, each with a duration of 1 min, and the results were expressed as the intensity-weighted mean diameter from multiple experiments. Zeta potentials were measured in ten runs, each consisting of ten cycles, using the Smoluchowski model. Furthermore, the hydrodynamic diameters of the nanoparticles were determined using nanoparticle tracking analysis (NTA) on an Insight LM 20 HS apparatus (Malvern, Worcester, UK) equipped with a 640 nm sCMOS camera and a temperature-controlled sample chamber, as previously described.

### 4.7. Assays for Silencing CTLA-4 In Vitro

In vitro CTLA-4 silencing assays were performed using qRT-PCR to study the activity of Exo-CTLA-4siRNA. 4T1 TNBC cells were seeded in a 12-well plate (2 × 10^5^ cells per well), and after 24 h, they were treated with Exo-CTLA-4siRNA (0.05 nmol siRNA), or PBS for 24 h. The cells were then washed with PBS, and total RNA was extracted using a Total RNA Kit (Norgen Biotek Corp, Thorold, ON, Canada). Reverse transcription was performed using HyperScript 1st Strand cDNA, and qPCR was carried out using Universal SYBR qPCR Mix. The mRNA levels of CTLA-4 were normalized to the endogenous housekeeping gene, GADPH.

### 4.8. In Vivo CTL Assay

Animal studies were conducted in accordance with institutional ethical guidelines. Sample size determination was based on preliminary data and calculated using G*Power 3.1.9.7. A minimum of six mice per group was required to detect a 30% difference in tumor volume with 80% power at a significance level of 0.05. Animals were randomly assigned to experimental groups using a random number generator, and tumor measurements were performed by an independent researcher blinded to treatment allocation.

The in vivo CTL assay was performed following a previously reported protocol with minor modifications. Briefly, female BALB/c mice were immunized with PBS without MUC1 mRNA (negative control). Ten days after immunization, splenocytes from naive mice were pulsed with lysates of 4T1 cells transfected with MUC1 mRNA in complete RPMI 1640 medium at 37 °C for 3 h. Only CFSE and PKH26 dyes were applied in the in vivo CTL killing assays, with no antibody staining of surface markers. Both dye concentrations and incubation times were precisely specified to ensure reproducibility.

### 4.9. Apoptosis Assays

In order to evaluate the effectiveness of Exo-CTLA-4siRNA as a therapeutic treatment, a 6-well plate was prepared with 4T1 TNBC cells (5 × 10^5^ cells per well) and incubated for 24 h. Subsequently, the cells were exposed to Exo-CTLA-4 siRNA (0.09 nmol siRNA), NLP-Exo-CTLA-4 siRNA (0.09 nmol siRNA), Exo (control), NLP (control), siRNA (0.09 nmol), and PBS for 48 h. To determine the effects of the treatment, Annexin V-FITC/PI was administered to the 4T1 TNBC cells, and their progress was monitored using flow cytometry.

### 4.10. Western Blotting

The procedure for preparing protein lysates from Tu2449 cells and mouse brain tumors was carried out according to published methods. SDS-PAGE and Western Blotting were also performed as described in the publication. To block the membranes, they were incubated in 5% BSA/TBS-Tween20 (TBS-T) at room temperature for 1 h. Antibodies were then added and incubated at 4 C overnight in 5% BSA/TBS-T. Secondary antibodies, either goat anti-mouse (dilution 5:20,000, PADTAN danesh, Tehran, Iran), were applied and incubated at room temperature for 1 h. For protein analysis of human cell cultures, the cells were seeded and transfected in 24-well plates as previously described. After 72 h, the medium was removed, and the cells were washed with PBS. RIPA lysis buffer (50 mM Tris (pH 7.4), 150 mM NaCl, 1% Triton X-100, 0.5% sodium deoxycholate, 0.1% SDS, 2.5 mM sodium pyrophosphate, 1 mM EDTA, Protease Inhibitor Cocktail Set III (EDTA-free, Merck, Darmstadt, Germany) was added to each well and the plates were incubated on ice for 10 min. The lysate was then transferred to microtubes and centrifuged at 10,000 rpm for 10 min at 4°C. The supernatant was collected, and protein concentration was determined using the TAKARA Protein-Assay (TAKARA, Kyoto, Japan). The protein lysate was mixed with 4 loading buffers (0.25 mM Tris-HCl, pH 6.8, 20% glycerol, 10% beta-mercaptoethanol, 8% SDS, 0.08% bromophenol blue) to yield a final concentration of 1 and 20 g was loaded onto 20% polyacrylamide gels. The proteins were separated by SDS-PAGE and transferred onto a 0.25 m Transfer PVDF Membrane (Millipore, Burlington, MA, USA). The membrane was then blocked with blocking buffer (5% (*w*/*v*) milk powder in TBST (10 mM Tris-HCl, pH 7.6, 150 mM NaCl, 0.1% Tween 20) for 40 min. Following a wash in TBST, membranes were treated with primary antibodies diluted in 3% milk powder (*w*/*v*) in TBST, including anti-human CTLA-4 (Thermo Fisher Scientific, Waltham, MA, USA), anti-Actin (Thermo Fisher Scientific), for an overnight incubation at 4 °C. The blots were then washed in TBST and exposed to horseradish peroxidase-coupled goat anti-rabbit IgG (CST) (Thermo Fisher Scientific) for 1 h in 3% milk powder (*w*/*v*) in TBST, followed by another wash. The presence of bound antibodies was detected using enhanced chemiluminescence (ECL) kits SuperSignal^®^ West Femto (Thermo Fisher Scientific).

### 4.11. Production of Mouse IFN-g Detected by ELISPOT Assay

Female BALB/c mice were immunized with nude NLE, PBS, and mRNA-loaded NLE, respectively. 10 days later, spleens were sterilely harvested from each treated mouse and separated into single cell suspensions. IFN-g production was measured with the BD ELISPOT assay system (BD PharMingen, San Jose, CA, USA) according to the manufacturer’s instructions. Briefly, cells were seeded at 1 × 10^6^ per well in a capture antibody-coated 96-well plate. The single cell suspensions were then cocultured with either 2 mg/mL 4T1 cell lysates transfected with MUC1 mRNA at 37 °C for 24 h Subsequently, cells were removed and the production of IFN-g was measured by adding a detection antibody, followed by an enzyme conjugate.

#### 4.11.1. Animal Experiments

Firstly, immune response enhancement was carried out. Female BALB/c mice received 1 × 105 4T1 tumor cells in the mammary fat pad on day 0. The NLE vaccine with mRNA, NLE vaccine with mRNA combined by CTLA-4-siRNA, nude NLE, and PBS were subcutaneously injected into the contralateral side of the lower flank on days 6 and 14, respectively. Tumor size was measured every 2 to 3 days using digital calipers (Thermo Fisher Scientific), and tumor volume was calculated as 0.5 × length × 2 × width.

#### 4.11.2. qRT-PCR

The amplification was carried out for 40 cycles with a pre-incubation at 95 °C for 15 s, followed by 10 s at 95 °C, 10 s at 55 °C, and 10 s at 72 °C for each cycle. A melting curve was obtained by rapidly cooling down from 95 °C to 65 °C, followed by a 15 s incubation at 65 °C and heating up to 95 °C. To normalize for equal mRNA/cDNA amounts, parallel runs were conducted with actin-specific primer sets for each sample. The ΔΔCt method was used to determine target levels. The following primer sets were used: human 4T1 cell line CTLA-4 forward primer: 5′ AGGTGACTGAAGTCTGTGCG 3′, reverse primer: TGATTTCCACTGGAGGTGCC; The following primer sets were used: Mus musculus CTLA-4 forward primer: 5′ CCGAGTCTGTGTGGGTTCAA 3′, reverse primer: GGTCCTCAGGGAGCAGAGTA; Samples from laser-capture-microdissected formalin-fixed paraffin-embedded (FFPE) tissues were combined, and RNA was extracted using the Arcturus^®^ Paradise^®^ PLUS FFPE RNA Isolation Kit (Thermo Fisher). The extracted RNA was then used to synthesize cDNA using SuperScript IV Vilo (life technologies/Thermo Fisher) following the manufacturer’s instructions. The qPCR was performed on a StepOne Plus System (Applied Biosystems, Darmstadt, Germany) using 20 Taqman Probes (Applied Biosystems, Darmstadt, Germany) and 2 Fast-Start Universal Probe Master Mix (Roche) with 2 µL of cDNA, using the standard setting. The gene expression values were normalized using the mean Ct value of two housekeeping genes, *Beta Actin* (*ACTB*) and *Glyceraldehyde-3-Phosphate Dehydrogenase* (*GAPDH*).

### 4.12. Statistics

Statistical analyses were conducted using GraphPad Prism version 7 (GraphPad Software, USA). The sample size for animal experiments was determined based on preliminary tumor volume data and an a priori power analysis using G*Power version 3.1.9.7. A minimum of 6 mice per group was required to detect a 30% difference in tumor volume with a power (1−β) of 0.80, a significance level (α) of 0.05, and an effect size (Cohen’s d) of approximately 1.5, assuming normally distributed data and equal variances. This sample size was deemed sufficient to detect biologically and clinically meaningful differences in immune response enhancement, CTL activity, and cytokine production in vivo. For comparisons between two independent groups, a two-tailed unpaired Student’s *t*-test was used, with the assumption of equal variances unless Levene’s test suggested otherwise. For experiments involving more than two groups or repeated measures across multiple conditions (e.g., cytokine expression or cell cycle distribution over time), two-way ANOVA with repeated measures was applied. For multiple comparisons, Dunnett’s test was used when comparing multiple treatment groups to a single control. When all group means were compared pairwise, Tukey’s post hoc test was employed to control the familywise error rate. In certain exploratory analyses, the Bonferroni correction was applied for more conservative control of Type I error, especially where multiple primary endpoints were assessed. Unless otherwise specified, data are presented as Box-and-Whisker plots, with the box indicating the interquartile range (IQR), the horizontal line denoting the median, and the whiskers extending to the minimum and maximum values. The calculated mean value is marked with an asterisk (*) in each plot. A *p*-value < 0.05 was considered statistically significant throughout the study, and significance levels were annotated as follows: * *p* < 0.05; ** *p* < 0.01; *** *p* < 0.001.

## 5. Conclusions

This study provides a first proof of concept for a co-delivery system using nano-lipid–exosome hybrids for the targeted delivery of MUC1 mRNA and CTLA-4 siRNA to enhance antitumor immunity in a TNBC model. While the MUC1-based mRNA vaccine elicited an antigen-specific cytotoxic T-cell response and enhanced T-cell infiltration into the tumor, the combination with CTLA-4 siRNA appeared to enhance this effect. These results suggest that the NLE platform has the potential for tumor-specific RNA delivery and immunomodulation. However, the data presented here should be interpreted as preliminary and exploratory. This work is a first step towards evaluating the therapeutic potential of RNA-based vaccines in combination with immune checkpoint elimination. Further extensive studies are required to investigate the safety, reproducibility, long-term immune effect and transferability of this approach to different tumor models and clinical scenarios.

In summary, this study presents a system that is distinctly different from similar studies in the literature by providing a dual immunotherapeutic approach through the combination of CTLA-4 siRNA and specific DC targeting enabled by Man-Nose-modified exosome-like lipid structures. The platform has been extensively characterized in terms of its physical, biochemical, and functional properties and shows high potential for clinical applicability.

## Data Availability

The dataset used during the current study is available from the corresponding author upon reasonable request.

## References

[1] Arnold M., Morgan E., Rumgay H., Mafra A., Singh D., Laversanne M., Vignat J., Gralow J.R., Cardoso F., Siesling S. (2022). Current and Future Burden of Breast Cancer: Global Statistics for 2020 and 2040. Breast.

[2] Beatson R., Maurstad G., Picco G., Arulappu A., Coleman J., Wandell H.H., Clausen H., Mandel U., Taylor-Papadimitriou J., Sletmoen M. (2015). The Breast Cancer-Associated Glycoforms of MUC1, MUC1-Tn and sialyl-Tn, Are Expressed in COSMC Wild-Type Cells and Bind the C-Type Lectin MGL. PLoS ONE.

[3] Obidiro O., Battogtokh G., Akala E.O. (2023). Triple Negative Breast Cancer Treatment Options and Limitations: Future Outlook. Pharmaceutics.

[4] Hargadon K.M., Johnson C.E., Williams C.J. (2018). Immune checkpoint blockade therapy for cancer: An overview of FDA-approved immune checkpoint inhibitors. Int. Immunopharmacol..

[5] Schmid P., Cortes J., Pusztai L., McArthur H., Kümmel S., Bergh J., Denkert C., Park Y.H., Hui R., Harbeck N. (2020). Pembrolizumab for early triple-negative breast cancer. N. Engl. J. Med..

[6] Nanda R., Chow L.Q., Dees E.C., Berger R., Gupta S., Geva R., Pusztai L., Pathiraja K., Aktan G., Cheng J.D. (2016). Pembrolizumab in Patients With Advanced Triple-Negative Breast Cancer: Phase Ib KEYNOTE-012 Study. J. Clin. Oncol..

[7] Bian L., Zhang H., Wang T., Zhang S., Song H., Xu M., Yao S., Jiang Z. (2019). JS001, an anti-PD-1 mAb for advanced triple negative breast cancer patients after multi-line systemic therapy in a phase I trial. Ann. Transl. Med..

[8] Emens L.A., Cruz C., Eder J.P., Braiteh F., Chung C., Tolaney S.M., Kuter I., Nanda R., Cassier P.A., Delord J.-P. (2019). Long-term clinical outcomes and biomarker analyses of atezolizumab therapy for patients with metastatic triple-negative breast cancer: A phase 1 study. JAMA Oncol..

[9] Jacobs F., Agostinetto E., Miggiano C., De Sanctis R., Zambelli A., Santoro A. (2023). Hope and hype around immunotherapy in triple-negative breast cancer. Cancers.

[10] Dirix L.Y., Takacs I., Jerusalem G., Nikolinakos P., Arkenau H.-T., Forero-Torres A., Boccia R., Lippman M.E., Somer R., Smakal M. (2018). Avelumab, an anti-PD-L1 antibody, in patients with locally advanced or metastatic breast cancer: A phase 1b JAVELIN Solid Tumor study. Breast Cancer Res. Treat..

[11] Kaewkangsadan V., Verma C., Eremin J.M., Cowley G., Ilyas M., Eremin O. (2018). Tumour-draining axillary lymph nodes in patients with large and locally advanced breast cancers undergoing neoadjuvant chemotherapy (NAC): The crucial contribution of immune cells (effector, regulatory) and cytokines (Th1, Th2) to immune-mediated tumour cell death induced by NAC. BMC Cancer.

[12] Sriramulu S., Thoidingjam S., Speers C., Nyati S. (2024). Present and Future of Immunotherapy for Triple-Negative Breast Cancer. Cancers.

[13] Liu Z., Chen J., Ren Y., Liu S., Ba Y., Zuo A., Luo P., Cheng Q., Xu H., Han X. (2024). Multi-stage mechanisms of tumor metastasis and therapeutic strategies. Signal Transduct. Target. Ther..

[14] Buonaguro L., Tagliamonte M. (2020). Selecting Target Antigens for Cancer Vaccine Development. Vaccines.

[15] Al-Aghbar M.A., Jainarayanan A.K., Dustin M.L., Roffler S.R. (2022). The interplay between membrane topology and mechanical forces in regulating T cell receptor activity. Commun. Biol..

[16] Hossen M.M., Ma Y., Yin Z., Xia Y., Du J., Huang J.Y., Huang J.J., Zou L., Ye Z., Huang Z. (2023). Current understanding of CTLA-4: From mechanism to autoimmune diseases. Front. Immunol..

[17] Kim G.R., Choi J.M. (2022). Current Understanding of Cytotoxic T Lymphocyte Antigen-4 (CTLA-4) Signaling in T-Cell Biology and Disease Therapy. Mol. Cells.

[18] Peng S., Lin A., Jiang A., Zhang C., Zhang J., Cheng Q., Luo P., Bai Y. (2024). CTLs heterogeneity and plasticity: Implications for cancer immunotherapy. Mol. Cancer.

[19] Rudd C.E., Taylor A., Schneider H. (2009). CD28 and CTLA-4 coreceptor expression and signal transduction. Immunol. Rev..

[20] Wu Y., Guo Y., Huang A., Zheng P., Liu Y. (1997). CTLA-4-B7 interaction is sufficient to costimulate T cell clonal expansion. J. Exp. Med..

[21] Beatson R., Tajadura-Ortega V., Achkova D., Picco G., Tsourouktsoglou T.D., Klausing S., Hillier M., Maher J., Noll T., Crocker P.R. (2016). The mucin MUC1 modulates the tumor immunological microenvironment through engagement of the lectin Siglec-9. Nat. Immunol..

[22] Bhatia R., Gautam S.K., Cannon A., Thompson C., Hall B.R., Aithal A., Banerjee K., Jain M., Solheim J.C., Kumar S. (2019). Cancer-associated mucins: Role in immune modulation and metastasis. Cancer Metastasis Rev..

[23] Dong S., Liang S., Cheng Z., Zhang X., Luo L., Li L., Zhang W., Li S., Xu Q., Zhong M. (2022). ROS/PI3K/Akt and Wnt/beta-catenin signalings activate HIF-1alpha-induced metabolic reprogramming to impart 5-fluorouracil resistance in colorectal cancer. J. Exp. Clin. Cancer Res..

[24] Matsushima N., Yoshida H., Kumaki Y., Kamiya M., Tanaka T., Izumi Y., Kretsinger R.H. (2008). Flexible structures and ligand interactions of tandem repeats consisting of proline, glycine, asparagine, serine, and/or threonine rich oligopeptides in proteins. Curr. Protein Pept. Sci..

[25] Supruniuk K., Radziejewska I. (2021). MUC1 is an oncoprotein with a significant role in apoptosis (Review). Int. J. Oncol..

[26] Al Fayez N., Nassar M.S., Alshehri A.A., Alnefaie M.K., Almughem F.A., Alshehri B.Y., Alawad A.O., Tawfik E.A. (2023). Recent Advancement in mRNA Vaccine Development and Applications. Pharmaceutics.

[27] Blakney A.K., Ip S., Geall A.J. (2021). An Update on Self-Amplifying mRNA Vaccine Development. Vaccines.

[28] Wang C., Yuan F. (2024). A comprehensive comparison of DNA and RNA vaccines. Adv. Drug Deliv. Rev..

[29] Wadhwa A., Aljabbari A., Lokras A., Foged C., Thakur A. (2020). Opportunities and Challenges in the Delivery of mRNA-Based Vaccines. Pharmaceutics.

[30] Cai X., Li J.J., Liu T., Brian O., Li J. (2021). Infectious disease mRNA vaccines and a review on epitope prediction for vaccine design. Brief. Funct. Genom..

[31] Li D.F., Liu Q.S., Yang M.F., Xu H.M., Zhu M.Z., Zhang Y., Xu J., Tian C.M., Yao J., Wang L.S. (2023). Nanomaterials for mRNA-based therapeutics: Challenges and opportunities. Bioeng. Transl. Med..

[32] Monfaredan A., Şen S., Hosseininasab A., Taştekin D., Fazli G., Bozbey H.U., Yousefi N., Hocaoğlu M., Öncül M.O., Özen R.S. (2025). Exosome Enveloped by Nano Lipid Particle a New Model for Signal Transducer and Activator of Transcription 3 Silencer Ribonucleic Acid Delivery System to a Glioblastoma Mice Model. Cancers.

[33] O’Brien J., Hayder H., Zayed Y., Peng C. (2018). Overview of MicroRNA Biogenesis, Mechanisms of Actions, and Circulation. Front. Endocrinol..

[34] Paurević M., Šrajer Gajdošik M., Ribić R. (2024). Mannose Ligands for Mannose Receptor Targeting. Int. J. Mol. Sci..

[35] Lin X., Chen H., Xie Y., Zhou X., Wang Y., Zhou J., Long S., Hu Z., Zhang S., Qiu W. (2022). Combination of CTLA-4 blockade with MUC1 mRNA nanovaccine induces enhanced anti-tumor CTL activity by modulating tumor microenvironment of triple negative breast cancer. Transl. Oncol..

[36] Liu Y., Zheng J., Liu Y., Wen L., Huang L., Xiang Z., Lam K.T., Lv A., Mao H., Lau Y.L. (2018). Uncompromised NK cell activation is essential for virus-specific CTL activity during acute influenza virus infection. Cell. Mol. Immunol..

[37] Pardi N., Hogan M.J., Porter F.W., Weissman D. (2018). mRNA Vaccines—A New Era in Vaccinology. Nat. Rev. Drug Discov..

[38] Sahin U., Tureci O. (2018). Personalized vaccines for cancer immunotherapy. Science.

[39] Zhao L., Seth A., Wibowo N., Zhao C.X., Mitter N., Yu C., Middelberg A.P. (2014). Nanoparticle vaccines. Vaccine.

[40] Zhang C., Maruggi G., Shan H., Li J. (2019). Advances in mRNA Vaccines for Infectious Diseases. Front. Immunol..

[41] Sharma P., Hu-Lieskovan S., Wargo J.A., Ribas A. (2017). Primary, Adaptive, and Acquired Resistance to Cancer Immunotherapy. Cell.

[42] Wei S.C., Duffy C.R., Allison J.P. (2018). Fundamental Mechanisms of Immune Checkpoint Blockade Therapy. Cancer Discov..

[43] Kranz L.M., Diken M., Haas H., Kreiter S., Loquai C., Reuter K.C., Meng M., Fritz D., Vascotto F., Hefesha H. (2016). Systemic RNA delivery to dendritic cells exploits antiviral defence for cancer immunotherapy. Nature.

[44] Suk J.S., Xu Q., Kim N., Hanes J., Ensign L.M. (2016). PEGylation as a strategy for improving nanoparticle-based drug and gene delivery. Adv. Drug Deliv. Rev..

[45] Liu M.A. (2019). A Comparison of Plasmid DNA and mRNA as Vaccine Technologies. Vaccines.

[46] Gao Y., Yang L., Li Z., Peng X., Li H. (2024). mRNA vaccines in tumor targeted therapy: Mechanism, clinical application, and development trends. Biomark. Res..

[47] Mitchell M.J., Billingsley M.M., Haley R.M., Wechsler M.E., Peppas N.A., Langer R. (2021). Engineering precision nanoparticles for drug delivery. Nat. Rev. Drug Discov..

[48] Xuan L., Ju Z., Skonieczna M., Zhou P.K., Huang R. (2023). Nanoparticles-induced potential toxicity on human health: Applications, toxicity mechanisms, and evaluation models. MedComm.

[49] Korotkov S.M. (2023). Mitochondrial Oxidative Stress Is the General Reason for Apoptosis Induced by Different-Valence Heavy Metals in Cells and Mitochondria. Int. J. Mol. Sci..

[50] Sousa de Almeida M., Susnik E., Drasler B., Taladriz-Blanco P., Petri-Fink A., Rothen-Rutishauser B. (2021). Understanding nanoparticle endocytosis to improve targeting strategies in nanomedicine. Chem. Soc. Rev..

[51] Mazumdar S., Chitkara D., Mittal A. (2021). Exploration and insights into the cellular internalization and intracellular fate of amphiphilic polymeric nanocarriers. Acta Pharm. Sin. B.

[52] Schoenmaker L., Witzigmann D., Kulkarni J.A., Verbeke R., Kersten G., Jiskoot W., Crommelin D.J.A. (2021). mRNA-lipid nanoparticle COVID-19 vaccines: Structure and stability. Int. J. Pharm..

[53] Li M., Zhu J., Lv Z., Qin H., Wang X., Shi H. (2024). Recent Advances in RNA-Targeted Cancer Therapy. Chembiochem.

[54] Kim C.-G., Sang Y.-B., Lee J.-H., Chon H.-J. (2021). Combining Cancer Vaccines with Immunotherapy: Establishing a New Immunological Approach. Int. J. Mol. Sci..

[55] Haabeth O.A.W., Blake T.R., McKinlay C.J., Waymouth R.M., Wender P.A., Levy R. (2018). mRNA vaccination with charge-altering releasable transporters elicits human T cell responses and cures established tumors in mice. Proc. Natl. Acad. Sci. USA.

[56] Zhou Z., Xu J., Liu S., Lv Y., Zhang R., Zhou X., Zhang Y., Weng S., Xu H., Ba Y. (2024). Infiltrating treg reprogramming in the tumor immune microenvironment and its optimization for immunotherapy. Biomark. Res..

[57] Guo S.B., Meng Y., Lin L., Zhou Z.Z., Li H.L., Tian X.P., Huang W.J. (2024). Artificial intelligence alphafold model for molecular biology and drug discovery: A machine-learning-driven informatics investigation. Mol. Cancer.

